# In Vivo Confocal Microscopy Characterization of *Candida parapsilosis* Keratitis

**DOI:** 10.1097/ICL.0000000000001059

**Published:** 2024-01-25

**Authors:** R. Scotto, P. Forte, A. Macrì, C. Bonzano, C. E. Traverso

**Affiliations:** Clinica Oculistica (R.S., P.F., C.B., C.E.T.), Dipartimento di Neuroscienze, Riabilitazione, Oftalmologia, Genetica e Scienze Materno-Infantili (DINOGMI), Università degli Studi di Genova, Genova, Italy; and IRCCS Ospedale Policlinico San Martino (P.F., A.M., C.B., C.E.T.), Genova, Italy.

**Keywords:** *Candida parapsilosis*, Keratitis, In vivo confocal microscopy

## Abstract

The present clinical case concerns two patients with mycotic keratitis because of *Candida parapsilosis* in which corneal confocal microscopy presented a characteristic feature of this pathogen. Both described patients used a therapeutic contact lens and administered a therapy with steroid eye drops which are well known predisposing factors for the onset of corneal mycoses. This report can be useful for correctly identifying the pathologic condition and quickly directing the therapy.

To characterize in vivo corneal confocal microscopy findings of a *Candida parapsilosis* keratitis.

*Candida parapsilosis* is a significant etiologic agent of keratomycosis and corneal graft fungal infection.^1^

Initial misdiagnosis before corneal scraping may lead to severe disease requiring surgical intervention, despite antifungal treatment.^2^

This opportunistic *Candida* species is a commensal on human skin and has been linked to outbreaks in the nosocomial setting.^3^

The fungal infection typically occurs on compromised corneas with a decrease of host defense, which has been described after laser in situ keratomileusis^4–6^ and corneal prosthesis.^7^

Clinical aspects of *Candida parapsilosis* keratitis show a delayed onset of symptoms and a variety of disease presentations, which include crystalline keratopathy, stromal corneal infiltrates, and suppurative keratitis.^8,9^

In vivo confocal microscopy (IVCM) is a valuable imaging modality that enables noninvasive, high-resolution, in vivo evaluation of corneal structures and pathologies at a cellular and subcellular level.^10–12^ IVCM has shown advantages in the early diagnosis of fungal keratitis, allowing a noninvasive and rapid guidance for an early initiation of treatment, with a relatively high sensitivity, specificity, and reproducibility for detecting fungal filaments.^13^

In this article, we present the specific in vivo corneal confocal microscopy findings of a *Candida parapsilosis* keratitis.

## CASE REPORT

### Case 1

A 92-year-old woman with bullous keratopathy presented to the University Eye Clinic of Genoa with unilateral painful vision loss and photophobia; visual acuity in her right eye was counting fingers. To reduce the symptoms of bullous keratopathy, the patient was wearing a therapeutic contact lens in association with the use of topical antibiotic–steroid combination. The patient did not report any history of diabetes or immunosuppression.

A complete ophthalmologic examination was performed, which included slit-lamp evaluation, anterior segment photography, anterior segment optical coherence tomography, IVCM (Heidelberg Retina Tomograph III-Rostock Cornea Module), and corneal scraping for fungal and bacterial cultures.

Biomicroscopy revealed diffuse conjunctival hyperemia and a large area of whitish non-homogeneous central corneal infiltration and the presence of satellite lesions. The margins of the lesions appeared jagged.

IVCM, using the field lens 300 μm (300×300 μm in 384×384 pixels), revealed the presence of numerous spherical infiltrates that show caviar-like appearance; comparing with the scale present in the IVCM images, the diameter of these lesions is approximately 2 μm.

**FIG. 1.**
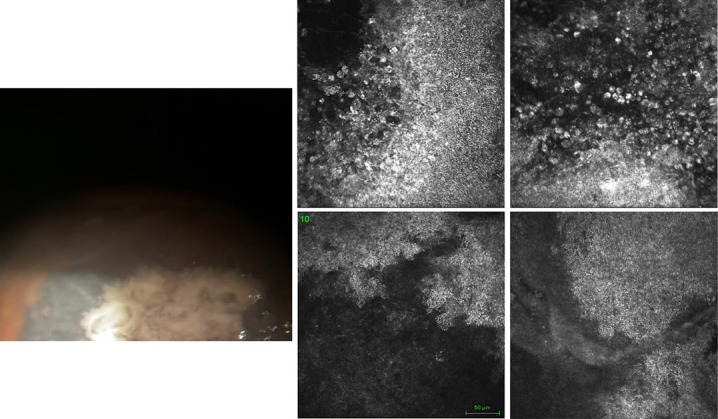
Case 1: The photograph shows the presence of rounded lesions, confluent with blurred margins, of whitish color. IVCM highlights the presence of large hyperreflecting areas formed by several spherical formations that show a caviar-like appearance, and their diameter, in some areas, gradually increases up to the edges of the lesion. IVCM, in vivo confocal microscopy.

The size of those lesions gradually increased up to the rim of the lesion, forming jagged edges as observed at the slitlamp (Fig. 1).

Guided by the suspect of a fungal keratitis topical voriconazole (1% drops) was initiated. Culture analysis of two separate corneal scraping and of the contact lens isolated the presence of *Candida parapsilosis*.

At 1-week examination, the lesion showed a favorable response to topical treatment, with a decrease of the corneal infiltrate and partial epithelialization of the cornea. A notable reduction was also documented in the number and density of the infiltrates as examined on control IVCM, that appeared as scattered hyperreflective microdots of little clinical significance. Visual acuity improved to 20/160.

**FIG. 2.**
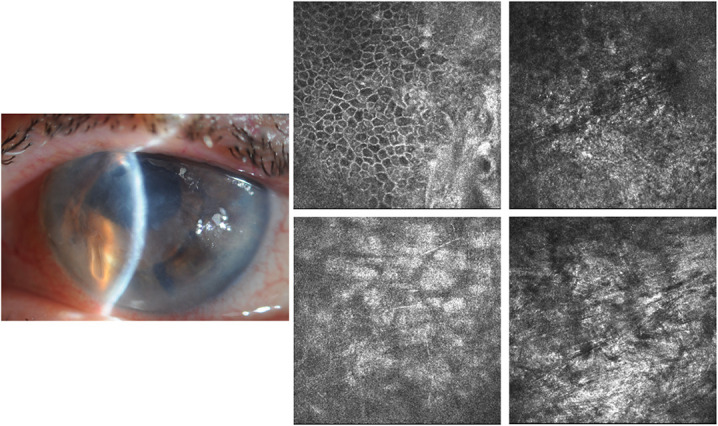
Case 1: After 1 month on slitlamp examination, the cornea presented marked improvement, whereas IVCM showed a total disappearance of the caviar-like infiltrates caused by the fungal infection. IVCM, in vivo confocal microscopy.

After 1 month, the patient was reevaluated and visual function remained stable, whereas the appearance of the cornea improved significantly with the complete disappearance of the infiltrates caused by the fungal infection (Fig. 2).

### Case 2

A 77-year-old woman was referred to the University Eye Clinic of Genoa for suspected graft rejection. She had five corneal transplants (three descemet stripping endothelial keratoplasty and subsequently two penetrating keratoplasty) in the right eye, initially for endothelial dystrophy and subsequently for graft rejection. The last penetrating keratoplasty had been performed in November 2022. The patient was wearing a therapeutic contact lens and instilling topical antibiotic–steroid combination every 2 hr and topical povidone–iodine (three times per day) and oral steroid treatment for about one month after what was suspected as another graft rejection.

A complete ophthalmologic examination was done, with slitlamp assessment, anterior segment photography, anterior segment optical coherence tomography, IVCM, and corneal scraping for fungal and bacterial cultures.

At the slitlamp, central whitish snowflake intrastromal infiltrates and rounded infiltrates with jagged edges in the periphery at the level of the graft sutures also located superficially with melted epithelium were observed. Endothelial deposits were present with no visible cells in the anterior chamber.

**FIG. 3.**
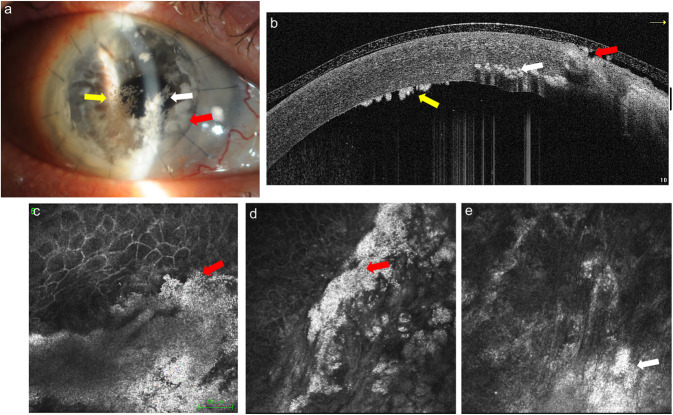
Case 2: The slitlamp and AS-OCT evaluation indicates central intrastromal whitish snowflake infiltrates (*white arrow*) and rounded infiltrates with jagged edges in the periphery at the level of the graft sutures (*red arrow*), located superficially with melted epithelium; also, endothelial deposits were present (*yellow arrow*) (a). The IVCM images emphasize lesions formed by spherical lesions similar to caviar, well-defined superficially (*red arrow*) (a–d), whereas at stromal level, the definition of white dots seems reduced (*white arrow*) (a–e). AS-OCT, anterior segment optical coherence tomography; IVCM, in vivo confocal microscopy.

IVCM (field lens 300 μm, 300×300 μm in 384×384 pixels) highlighted the presence of spherical lesions (diameter approximately 2 μm) similar to caviar, well-defined especially where the lesions were more superficial (Fig. 3).

We hypothesized a *Candida parapsilosis* keratitis discontinued the use of corticosteroids and started topical voriconazole and added systemic antifungal therapy with fluconazole. The culture of scraping confirmed *Candida parapsilosis* keratitis.

## DISCUSSION AND CONCLUSIONS

This report aims to describe the features of *Candida parapsilosis* keratitis observed by IVCM, which can be useful to make an early diagnosis and start quick an appropriate therapy.

To date, IVCM has mainly been used in the assessment of filamentous fungi and *Acanthamoeba* keratitis.^11,14^ Unlike *Candida albicans* and *Candida tropicalis*, which can exist in multiple morphogenetic forms, *Candida parapsilosis* does not form true hyphae and its yeast cells typically display oval or round shape.^15,16^

Interestingly, the caviar-like appearance of the yeast infiltrate by IVCM matches the histologic aspect of oval Gomori methenamine silver–positive fungal elements described in previous reports.^17,18^ The pathologic condition of graft specimens reported by Bourcier et al.^8^ displayed the similar presence of oval PAS-positive fungal organisms growing between stromal lamellae.

IVCM images of *Candida albicans* infection appears with a rounded shape and similar dimensions to *Candida parapsilosis* but pseudohyphae are also frequently present.^13,16,19^

The reproducibility of the present findings has to be assessed in future reports. However, the specific morphology we report shows great potential to lead timely initiation of antifungal treatment of this potentially sight-threatening infection, favoring a positive clinical outcome.

It is likely that both of our cases of *Candida parapsilosis* were triggered by the wear of soft therapeutic contact lenses and by the long-term use of topical corticosteroids and antibiotics, well-known predisposing factors to the onset corneal yeast infection.
